# Investigating the Effect of Temperature History on Crystal Morphology of Thermoplastic Composites Using In Situ Polarized Light Microscopy and Probabilistic Machine Learning

**DOI:** 10.3390/polym15010018

**Published:** 2022-12-21

**Authors:** Mathew Wynn, Navid Zobeiry

**Affiliations:** Materials Science & Engineering Department, University of Washington, Seattle, WA 98195, USA

**Keywords:** thermoplastic composites, processing, crystallinity, polarized light microscopy, machine learning

## Abstract

Processing parameters including temperature history affect the morphology of semicrystalline thermoplastic composites, and in turn their performance. In addition, the competition between spherulite growth in resin-rich areas, and transcrystallinity growth from fiber surfaces, determines the final morphology. In this study, growth of crystals in low volume fraction PEEK-carbon fiber composites was studied in situ, using a polarized microscope equipped with a heating and cooling controlled stage and a probabilistic machine learning approach, Gaussian Process Regression (GPR). GPR showed that for spherulites, growth kinetics follows the established Lauritzen-Hoffman equation, while transcrystallinity growth deviates from the theory. Combined GPR model and Lauritzen-Hoffman equation were used to deconvolute the underlying competition between diffusion and secondary nucleation at growth front of spherulites and transcrystalline regions.

## 1. Introduction

Thermoplastic composites offer excellent mechanical properties such as high impact resistance, as well as enhanced environmental and chemical resistance compared to amorphous thermoplastics. This, in addition to the promise of shorter processing times and reprocessability, has led to a surge of applications for thermoplastic composites in recent years. Those fabricated with high-performance semi-crystalline thermoplastics such as PEEK (Polyetheretherketone), PEKK (Polyetherketoneketone) and LM-PAEK (Low melt Polyaryletherketone) are of particular interest in aerospace industry. Examples include PEEK composite used in the international space station mechanical arm, or brackets of commercial aircrafts [1,2]. For such applications, thermoplastic composites are typically consolidated and processed using manufacturing techniques such as automated fiber placement (AFP) followed by autoclave consolidation or compression molding. In these processing methods, parameters such as melt temperature history, dwell time, fiber volume fraction as well as shear deformation history highly affect the crystallinity of the end-part [3,4,5]. Without proper crystallization, these thermoplastics typically tend towards their amorphous properties offering much lower mechanical properties as well as little resistance to chemicals.

Even with similar degree of crystallinity, crystal types and crystalline morphology may affect the performance of composite parts [6,7]. The primary structure formed in PEEK is the spherulite with lamellae radiating from a center nucleus with amorphous polymer chains filling the spaces in between [8]. The lamellae are comprised of folded chains that align perpendicular to the growth front. The crystallization process is governed by nucleation and growth where after initial nuclei have formed in the supercooled melt, the crystal goes through the growth process. There have been many attempts in the literature to describe the crystallization growth kinetics [9]. One of the most common models is Lauritzen-Hoffman theory, where growth is governed by a competition between the rate of secondary nucleation and rate of diffusion [10,11]. During secondary nucleation, nuclei are constantly formed onto the surface of a primary parent crystal. Here, primary nucleation is differentiated as the initial nucleation from melt, and secondary nucleation occurs from the surfaces of primary nuclei. However, molecular-level details of this process is not well understood [12]. On the other hand, diffusion is the transportation process of polymer segments and hence lateral growth along the crystallization front [9,11,12].

When fibers are present, a second type of crystalline structure can form, known as transcrystallinity [13]. This occurs when crystal lamellae directly nucleate from the fibers and grow primarily perpendicular to them [14]. For high fiber volume fraction composites, transcrystallinity dominates the morphology, while in resin rich areas such as in gaps and overlaps created by AFP processing, spherulites dominate the microstructure due to lack of heterogeneous sites. At the boundary of these regions, the competition between nucleation and growth of these crystals determines the final morphology and hence material performance. Such morphology highly depends on the processing conditions including temperature history. For example, a sufficiently high melting temperature inhibits PEEK’s tendency for self-nucleation by removing thermal history, which increases the likelihood to form transcrystallinity in PEEK composites [15]. PEEK also has a tendency for secondary crystallization [16,17]. This occurs when a lower melting temperature crystal structure forms on reheat, typically during annealing after initial crystallization [18]. The growth rate and dimensions of transcrystalline regions are typically similar to spherulites [19].

**Figure 1:**
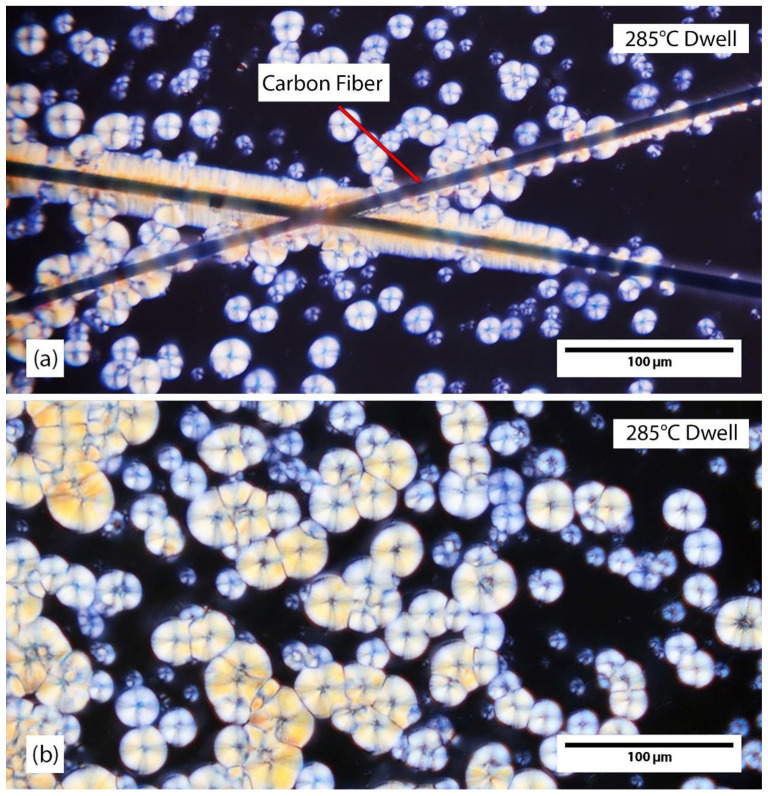
Examples of transcrystallinity and spherulites after a 285 °C crystallizing dwell (**a**) shows a transcrystalline region growing from both fibers, with a spherulitic-type growth occurring on the fiber in the top right. Spherulites are observed in the surrounding material. (**b**) shows spherulites of varying sizes, with some impinging onto others and others isolated and still circular.

Examples of transcrystallinity and spherulites are provided in Figure 1. In Figure 1a, transcrystallinity is seen on both fibers. One transcrystalline region is primarily a uniform growth perpendicular from the fiber. The other region is mostly spherulitic in nature. The spherulitic type transcrystalline region is an intermediate form. With further time at temperature, the spherulitic growths from the fiber will likely fill out and become more like the other transcrystalline region shown in Figure 1a. Circular spherulites of varying sizes can be seen in both Figure 1a,b. When spherulites grow and impinge upon each other, growth stops at that interface and the spherulite becomes non-circular. The crystals shown in Figure 1 have been stopped mid-growth and the black region in between crystals is amorphous polymer yet to be crystallized. Typically for PEEK, the entire surface will be covered with crystals unless quenched. In addition, the transcrystalline regions and spherulites shown in Figure 1 are not stacked. This was confirmed during the cooldown of this sample, which was observed in real-time.

To understand the effects of processing parameters on microstructure, characterization methods such as DSC or Wide-angle X-ray Scattering (WAXS) can be utilized to measure the degree of crystallinity as a homogenized macro property [20,21]. These methods, however, do not reveal types and gradients of microstructures within the material. For a direct characterization approach, polarized light microscopy (PLM) is often used [5,8,22]. The crystalline portion of the microstructure exhibits birefringence which allows for microstructure characterization through transmission PLM [23]. Regions that go to extinction in a crossed-polarized light are non-crystalline. These are either amorphous polymer, oxidized cross-linked polymer, or fibers. Other microscopic methods are possible, such as etching and scanning electron microscopy (SEM) or atomic force microscopy (AFM) [24,25]. SEM is limited to the effectiveness of etchant and surface evaluations, while AFM provides an indirect perspective based on sample compliance with limited scanning area. In addition to difficulties with sample preparation, there also are challenges with data reduction and analysis. Specifically, fitting kinetics models for nucleation, growth, and melting of crystals using visual methods such as microscopy is not trivial. Due to testing and material variabilities, the data from these methods have a high degree of noise, and is affected by measurement errors. Considering these challenges, a robust and accelerated method is needed to evaluate the effects of various processing parameters on crystallization kinetics and final crystalline morphology of thermoplastic composites.

In recent years, Machine Learning (ML) models have been successfully utilized for a variety of composites applications including predicting shimming during assembly [26], detection of defects during automated fiber placement [27], damage characterization [28,29,30,31], and real-time process simulation [32,33]. Between all different ML approaches, probabilistic machine learning techniques such as Gaussian Process Regression (GPR) have received much attention due to their capability of analyzing small datasets that are corrupted by noise and errors [34]. GPR is a non-parametric ML approach to regression, where it can identify the underlying correlation in data while quantifying uncertainty of predictions. This makes this approach quite suitable for analyzing noisy and high-dimensional small datasets.

In this study, we present an accelerated method to study the effect of temperature cycle on crystalline morphology of thermoplastic composites using combined in situ PLM and GPR modeling. Initially, thin samples of low volume fraction composites are prepared for in situ study of crystallinity using a PLM equipped with a heating and cooling controlled stage. This enables studying both bulk-formed spherulites and transcrystallinity originating from fibers, as well as the competition for nucleation and growth. Using this approach, PEEK-carbon fiber composite samples were processed under a PLM subjected to four different temperature cycles. Spherulite and transcrystallinity growth rates were directly measured from microscopy videos. GPR was used to fit growth kinetics of both crystal types with high confidence. This showed that while spherulite growth kinetics follows the Lauritzen-Hoffman equation, transcrystallinity deviates from the theory. This was further studied by deconvoluting kinetics to underlying competition between diffusion and secondary nucleation.

## 2. Material and Methods

**Figure 2:**
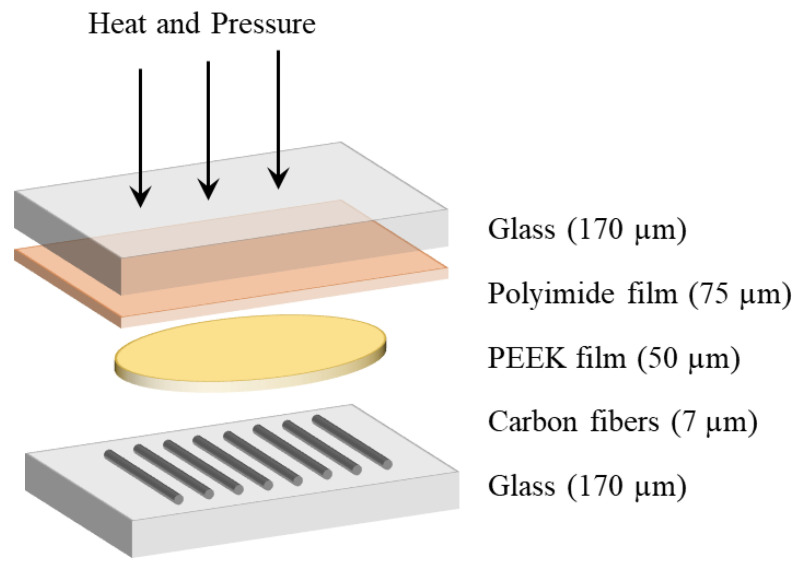
Sample stack-up used to produce thin PEEK samples with heat and pressure in a DMA.

A method was developed to prepare thin and low volume fraction composite films of PEEK resin and carbon fibers to conduct in situ PLM during crystallization. As schematically depicted in Figure 2, initially a piece of 75 µm thick PEEK film (Victrex, Thornton-Cleveleys, UK) was placed on top of heat cleaned HexTow AS4 carbon fibers (Hexcel, Stamford, CT, USA), sandwiched between 170 µm glass cover slips and a piece of polyimide film (Kapton, DuPont, Wilmington, DE, USA) coated with silicone-based Frekote 710-NC mold release (Henkel, Düsseldorf, Germany). 7 μm diameter carbon fibers were heat cleaned at 350 °C for 30 min to remove sizing while minimizing any damage [35]. Polyimide film was used as it is stable at the melting temperature of PEEK. When this polyimide is combined with Frekote, it prevents bonding to the glass. This is necessary to ensure sufficiently large crystals grew to allow measurement in PLM. When the PEEK sample is bonded to two pieces of glass, nucleation was assumed to be more frequent leading to small crystals that could not be measured in PLM. Samples were then placed into a compression fixture of a DMA 850 from TA Instruments (New Castle, DE, USA), and heat and pressure were applied in air. The stack-up was heated to 320 °C and held for 20 min to stabilize, before heating to 400 °C for 10 min. The heated 0.2 mm^2^ sample was compressed using 18 N of force applied over one minute. The resulting samples were found to be 20 ± 3 μm thick using a Bruker DekTak profilometer (San Jose, CA, USA). The fiber volume fraction was estimated at 11% using image analysis of micrographs of samples. This was kept low to allow for simultaneous spherulite and transcrystallinity growths in samples.

**Figure 3:**
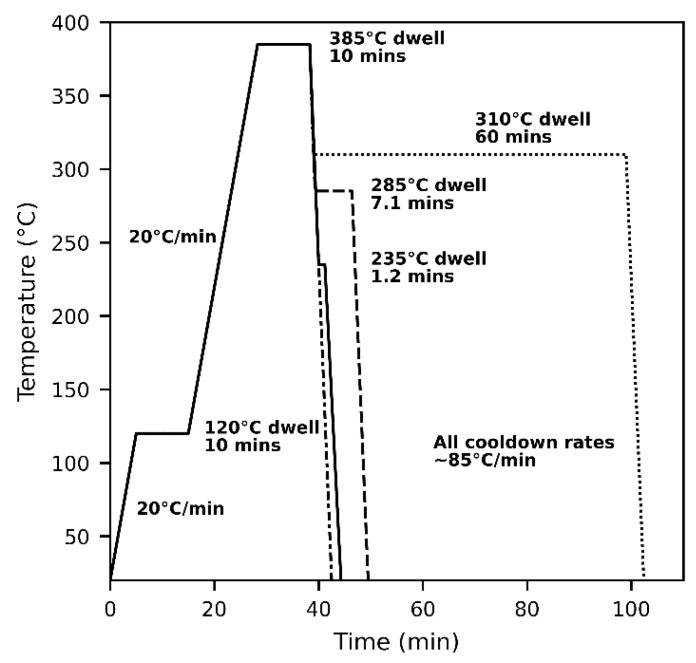
Heat cycle used for four processes: Three isothermal dwells at 310 °C, 285 °C, and 235 °C and one constant cooldown at 85 °C/min. Dwells were held until full crystallization.

The compressed samples were then heated to 420 °C at 20 °C/min and held for 10 min in an inert atmosphere of nitrogen using a Linkam THMS600 hot stage (Redhill, UK) to remove history from DMA processing, primarily due to polymer alignment or shear effects arising from compressing the sample. The samples were cooled down as fast as possible using the THMS600 at around 125 °C/min to minimize crystallization on cooldown. In addition, this 420 °C pretreatment slows down nucleation and growth in subsequent heating cycles, and this is likely due to cross-linking and oxidation that occurs at high melt temperatures in PEEK. Slowing nucleation and growth in this way is necessary to allow for repeatable measurement of crystal growth rate and size. These heat-treated samples were then processed again in a nitrogen environment using the Linkam hot stage. An Olympus BX61 (Tokyo, Japan) polarizing light microscope (PLM) with crossed polarizers was used to monitor samples as they were melted and recrystallized. Videos were recorded with a 24 MP digital camera (Sony α6000, Tokyo, Japan) installed on the microscope. Samples were heated to 120 °C for 10 min to remove moisture, and then heated at 20 °C/min to 385 °C to melt for 10 min. A 385 °C melting temperature was chosen as it is the manufacturer recommended melt temperature that minimizes degradation while maximizing removal of prior thermal history. Some thermal history may remain at this point, but this study chose to minimize degradation while ensuring artificial changes induced by DMA compression were removed. As shown in Figure 3, effects of four temperature cycles during cool-down on crystallinity were studied using in situ PLM: a 310 °C isothermal dwell, 285 °C dwell, 235 °C dwell, and a constant cooldown at 85 °C/min to room temperature. Each dwell was held until the surface was fully crystallized as observed in the microscope, after which the samples were cooled down at 85 °C/min. The resulting dwell for 285 °C was 7.1 min, and for 235 °C was 1.2 min. The 310 °C dwell was stopped after 60 min without completely crystallizing the whole surface before cool-down. Videos taken during melt and recrystallization were used to measure growth rate. Transcrystallinity and spherulites were selected such that they were segregated from other crystalline features. Measurements were taken before impingement occurred to minimize the effect on growth rate.

## 3. Results

### 3.1. Polarized Light Microscopy

**Figure 4:**
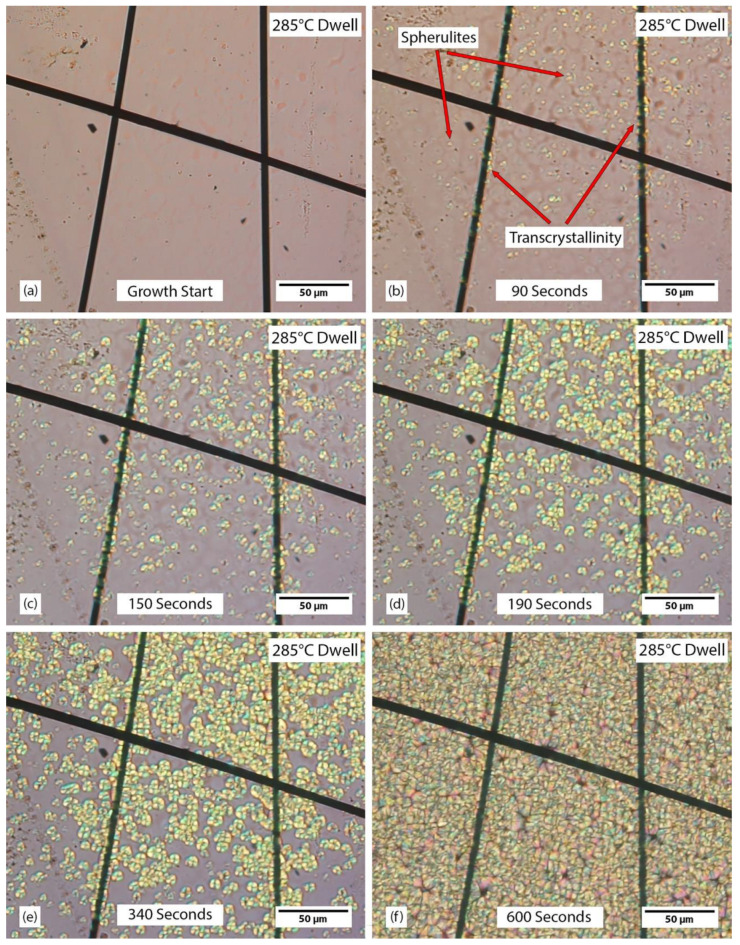
Time lapse growth of spherulites and transcrystallinity during a 285 °C dwell (**a**) molten polymer (**b**) nuclei start forming in the bulk polymer and from the fibers. (**c**,**d**) growth continues (**e**) transcrystallinity stops growth (**f**) visible growth stops.

An example of spherulite and transcrystalline growth during a 285 °C isothermal dwell is shown in Figure 4, which is a series of frames from a video taken in situ. Figure 4a,f show before and after crystal growth, respectively. Figure 4b–f shows crystal growth with increasing time: 0 s, 90 s, 150 s, 190 s, 340 s, and 600 s. Transcrystallinity can be seen nucleating in Figure 4b and grows until visible growth stops in Figure 4f. This was determined by analyzing the entire source video for the frames shown in Figure 4. This transcrystalline growth begins spherulite-like similar to one of the fibers shown in Figure 3. After some time, the growth transitions to be more traditional transcrystallinity. The transition from spherulite-like to transcrystallinity indicates an increase of nucleation along the fiber. An alternative method of transcrystallinity formation involves an excess of initial nuclei across the fiber, essentially skipping the spherulite-like phase. As initial nuclei cannot be detected with the resolution of this PLM technique, it is not clear whether spherulites or transcrystallinity nucleated first.

**Figure 5:**
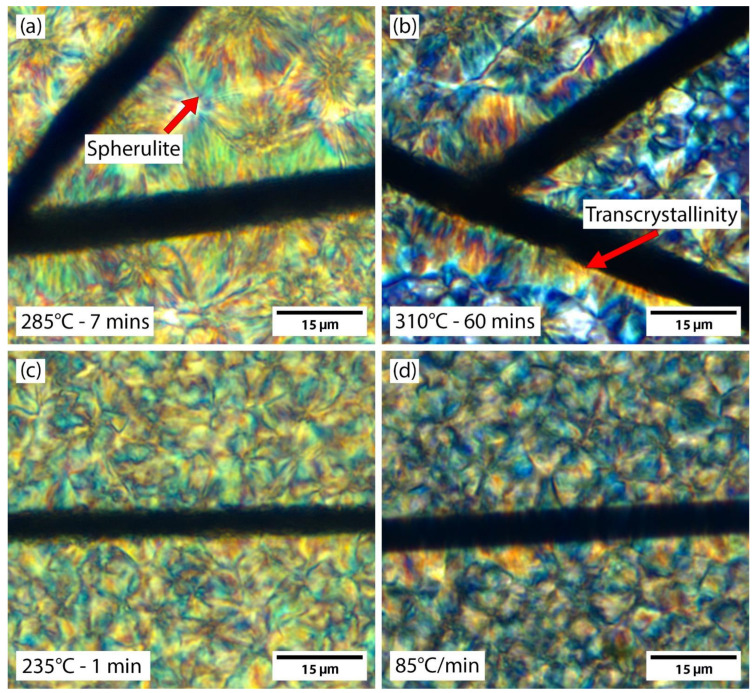
Morphology changes in each process. (**a**) a 285 °C isothermal dwell produces larger transcrystalline regions and large spherulites. An example spherulite is identified. (**b**) the 310 °C dwell produces the largest spherulites and transcrystalline regions. An example transcrystalline region is identified. (**c**) a 235 °C dwell produces minor transcrystalline growth and small spherulites that do not resolve in PLM. (**d**) an 85 °C/min cooldown shows minimal transcrystalline growth and spherulites do not resolve.

A comparison of the final microstructures in all four cycles is shown in Figure 5. Data for spherulite and transcrystalline growth rates are summarized in Table 1. When measuring size to calculate growth rate, spherulite diameter and double the transcrystallinity length normal to the fiber surface are used. The 285 °C and 310 °C isothermal dwells, shown in Figure 5a,b, produced the largest spherulites and transcrystalline regions. The 235 °C dwell and 85 °C/min cooldown as shown in Figure 5c,d produced small spherulites that did not have clear edges in PLM. This may be due to the limitation of the light microscopy technique. There was slight transcrystalline growth at 235 °C, and more consistent transcrystalline growth after an 85 °C/min cooldown. To fully resolve the smaller transcrystalline regions in Figure 5c,d, carbon fibers were put out of focus, making them look smaller than Figure 5a,b. All samples had a large amount of variation in growth rate data as listed under Table 1.

### 3.2. Growth Rate Analysis

**Figure 6:**
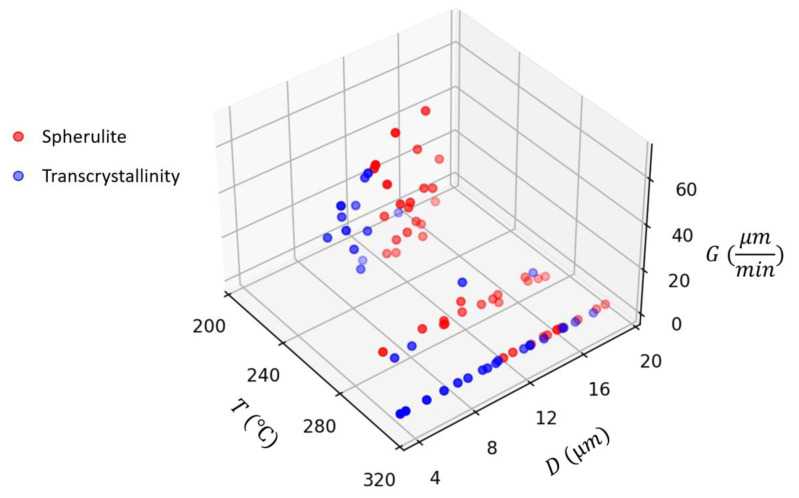
Growth rate data (*G*) measured from microscopy videos at different temperatures (*T*) and for different crystal sizes (*D*).

As mentioned earlier, growth rates were measured from in situ microscopy videos for both spherulites and transcrystalline regions. Measurements of growth rates, *G*, are shown in Figure 6 as a function of temperature, *T*, and crystal size, *D*. Mean and standard deviation data are provided in Table 1. From these results, it can be observed that the growth rate was fastest in samples with high undercooling, specifically the 235 °C and 85 °C/min cycle samples. These samples had the largest variation in growth rate as well. Based on mean data, transcrystallinity typically grew slower than spherulites. This may be attributed to the growth of nearby spherulites to slow down transcrystallinity growth. There is some error in identifying the edges of spherulites and transcrystalline regions. As the crystalline feature approaches the pixel size, it becomes increasingly difficult to accurately find the edges. To minimize the measurement errors, care was taken to only measure spherulites and transcrystalline regions where no impinging had occurred and were clearly separated from others. There were 3.76 pixels/μm in our microscopy images. While smallest measured spherulite had a diameter of about 3.7 μm, the largest was about 21.4 μm. However, most measured features were between 5 to 15 μm. Based on this, and assuming ±2 pixels error in measurements, the average estimated error for all measurements was about 6%.

In the first step, spherulite and transcrystalline growth rate data were fitted to the Lauritzen-Hoffman equation below [10,11]:

(1) $$G=G_{0}\;\exp\left[\frac{-U^{*}}{R\left(T_{c}-T_{\infty }\right)}\right]\exp\left[\frac{-K_{g}^{G}}{T_{c}\Delta Tf}\right]$$

where the first exponential term describes the contribution of the diffusion process to growth, and the second exponential term is associated with secondary nucleation. Here, G is the growth rate, $G_{0}$ is a growth rate constant, $U^{*}$ is the activation energy for diffusion across melt and crystal boundary (2800 J/mol [36]), R is the universal gas constant, $T_{\infty }$ is 30 °C less than glass transition temperature, $T_{g}$ (143 °C for PEEK), $T_{c}$ is the isothermal crystallization dwell temperature, $K_{g}^{G}$ is a parameter that is proportional to the energy barrier for secondary nucleation, $\Delta T=T_{m}^{0}-T_{c}$, $T_{m}^{0}$ is the equilibrium melting temperature (380.5 °C [36]), and *f* is a correction factor equal to $2T_{c}/\left(T_{m}^{0}+T_{c}\right)$. The logarithmic transformation of Equation (1) results in the following relationship:

(2) $$\ln\left(G\right)+\frac{U^{*}}{R\left(T_{c}-T_{\infty }\right)}=\;\ln\left(G_{0}\right)-\frac{K_{g}^{G}}{T_{c}\Delta Tf}$$

where the left side expression is linearly correlated to $1/\left(T_{c}\Delta Tf\right)$. Based on measured growth rates, initially a linear fit was found for spherulite growth following Equation (2), however, transcrystallinity deviated from the Lauritzen-Hoffman equation and showed a nonlinear correlation. To better understand these underlying correlations for different crystal types, GPR regression was used. For GPR training, a kernel or covariance matrix, Σ, is selected to describe correlations between datapoints in the observation domain. Given *n* measurements of $Y_{n}$ for *n* inputs of $X_{n}$ (i.e., prior), mean and variance functions, $\mu \left(X\right)$ and $\sigma^{2}\left(X\right)$, in the desired domain (i.e., posterior) can be calculated as [37]:

(3) $$\mu \left(X\right)=\Sigma \left(X,X_{n}\right)\Sigma^{-1}\left(X_{n},X_{n}\right)Y_{n}$$

(4) $$\sigma^{2}\left(X\right)=\;\Sigma \left(X,X\right)-\Sigma \left(X,X_{n}\right)\Sigma^{-1}\left(X_{n},X_{n}\right)\Sigma (X_{n},X)$$

To take advantage of existing theory, a transformed dataset based on Lauritzen-Hoffman equation was used for GPR training with dimensions of :

(5) $$X=\left(\frac{1}{T_{c}\Delta Tf},\;D\right)\;\mathrm{and}\;Y=\ln\left(G\right)+\frac{U^{*}}{R\left(T_{c}-T_{\infty }\right)}$$

**Figure 7:**
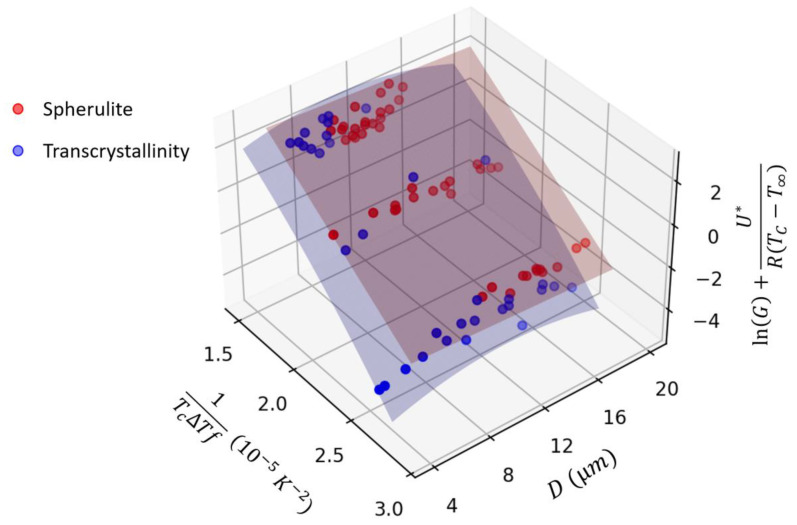
Fitted GPR models to spherulite and transcrystallinity growth rate data in the transformed Lauritzen-Hoffman domain (Equation (2)).

GPR training was performed using the scikit-learn library in Python. Data was split into 80% training data and 20% validation data. For spherulite growth, following Lauritzen-Hoffman theory, a combination of linear white noise kernels was initially selected:

(6) $$\Sigma \left(x_{i},x_{j}\right)=\;c\times \left[\sigma_{0}^{2}+x_{i}\cdot x_{j}\right]+\sigma_{e}^{2}I_{n}$$

where c is a constant, $\sigma_{0}^{2}$ is a constant controlling the inhomogeneity of the linear kernel, $\sigma_{e}^{2}$ is the variance of noise, and $I_{n}$ is the identify matrix. In this study, default values of $\sigma_{0}^{2}$ and $\sigma_{e}^{2}$ equal to unity were used. Based on this linear correlation, trained GPR model showed an accuracy of 96% for the validation data. The fitted mean response surface from GPR model is shown in Figure 7. For transcrystallinity, initially a kernel similar to Equation (6) was selected; however, this only showed an accuracy of 88% for the validation set. After this first trial, a second kernel was selected as a combination of the square of the linear kernel and white noise kernel:

(7) $$\Sigma \left(x_{i},x_{j}\right)=\;c\times {\left[\sigma_{0}^{2}+x_{i}\cdot x_{j}\right]}^{2}+\sigma_{e}^{2}I_{n}$$

Upon training, GPR showed an accuracy of 98% to predict the validation dataset. The fitted mean response surface for transcrystallinity growth is shown in Figure 7. This figure clearly shows that while spherulite growth follows a linear correlation as predicted by Lauritzen-Hoffman Equation (2), transcrystallinity shows a nonlinear correlation. From these fitted GPR models, energy barriers for secondary nucleation in Lauritzen-Hoffman equation ($K_{g}^{G}$) can be estimated to be 4.7 (10^5^ K^2^) for spherulites and 5.5 (10^5^ K^2^) for transcrystallinity. From these estimated values (also confirmed in Figure 7), it can be observed that growth rates are in general faster for spherulites compared to transcrystallinity regions.

## 4. Discussion

**Figure 8:**
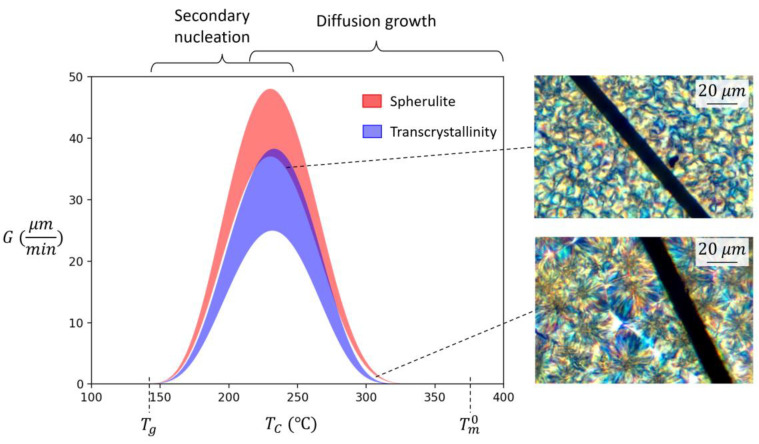
Comparison of growth rate as a function of temperature for spherulitic and transcrystallinity growths. Examples of final morphologies at different temperatures are superposed on the graph.

The in situ PLM method enables direct analysis of nucleation, growth, and final crystalline morphology as a function of processing parameters. Additionally, GPR fitting combined with the Lauritzen-Hoffman equation provides insights into the nature of the competing mechanisms during crystallization. The first exponential term in Equation (1) represents diffusion growth as a function of $T_{C}-T_{\infty }$, which is similar for both spherulite and transcrystallinity growth. However, the second exponential term represents secondary nucleation as a function of $T_{C}\left(T_{m}^{0}-T_{C}\right)$, and is different for spherulite and transcrystallinity due to different energy barriers for secondary nucleation as estimated in the previous section. Fitted GPR models were used to calculate and plot growth rate as a function of temperature in Figure 8 for both spherulites and transcrystallinity regions. This further confirms that compared to spherulites, transcrystallinity has slower growth rates at similar undercooling temperatures [9,38].

Another important observation is the bell-shaped curves in Figure 8. While diffusion growth increases closer to $T_{m}$, secondary nucleation increases closer to $T_{g}$. The combination of these two mechanisms leads to a maximum growth rate somewhere in between the glass transition and melting temperatures, where thermal energy for diffusion is abundant and the drive for secondary nucleation high. In addition, the small undercooling at temperatures close to the melting temperature reduces the quantity of initial nuclei, leading to large spherulites and transcrystallinity regions as demonstrated by PLM results in Figure 5a,b, and also in Figure 8. At temperatures close to the glass transition temperature, small spherulites and transcrystallinity regions are formed with unclear boundaries as shown in Figure 5c and Figure 8 due to high drive for initial nucleation but relatively low thermal energy for growth.

With transcrystallinity, a high amount of initial nucleation occurs along the fiber as seen in Figure 1. Most of the transcrystallinity observed in this study was spherulitic in nature, indicating initial nucleation was infrequent along the carbon fiber. The extent of this nucleation can be limited by the growth of neighboring spherulites [39]. Since the transcrystalline region is comprised of the same lamellar structure as spherulites, in theory, its growth rate should follow the Lauritzen-Hoffman equation. In this study, however, nonlinear growth kinetics are observed in GPR fitting (Figure 7), deviating from the Lauritzen-Hoffman equation. Additionally, transcrystalline growth shows correlation to the size of transcrystalline region (*D*). Based on Equation (1), size should not affect secondary nucleation. This, however, can be explained based on the competition between spherulite and transcrystallinity growth. As discussed earlier and evident in Figure 8, spherulite growth rate is faster compared to transcrystalline regions. As such, spherulites may hinder the growth of transcrystallinity, depending on their size. This potentially can create nonlinearity in Lauritzen-Hoffman domain, and hence dependency of growth to the crystal size.

## 5. Conclusions

Deconvolution of growth kinetics for both spherulite and transcrystallinity regions showed the underlying competition between secondary nucleation and diffusion at different undercooling temperatures. At temperatures closer to the melting temperature, growth was dominated by lateral diffusion. At temperatures closer to glass transition temperature, growth was dominated by secondary nucleation.

While spherulite growth follows the Lauritzen-Hoffman equation with a linear correlation in the logarithmic domain, transcrystalline growth was found to exhibit nonlinearity, deviating from the Lauritzen-Hoffman equation. This was attributed to the impinging effect of spherulites growing near fibers. Machine learning fitting using GPR showed a quadratic correlation for transcrystallinity growth.

In general, spherulites grow faster at similar undercooling temperatures compared to transcrystallinity. This was attributed to a larger energy barrier for secondary nucleation at the transcrystalline growth front.

Finally, this study demonstrated the effective application of GPR method as a probabilistic machine learning approach for analyzing small experimental datasets corrupted by noise. The combination of GPR with established theory for crystallization provided a deeper understanding of the competing nature of underlying mechanisms.

## Figures and Tables

**Table 1:** Growth rate data measured from microscopy videos for spherulites and transcrystallinity regions.

Heating Cycle	Spherulite (µm/min)		Transcrystallinity (µm/min)	
	Average	Standard Deviation	Average	Standard Deviation
310 °C	1.0	0.2	0.4	0.2
285 °C	8.1	2.1	10.1	7.4
235 °C	40.0	12.2	37.8	13.5
85 °C/min	41.7	19.0	32.5	12.6

## Data Availability

The data presented in this study are available on request from the corresponding author.

## References

[1] Denault J., Dumouchel M. (1998). Consolidation Process of PEEK/Carbon Composite for Aerospace Applications. Adv. Perform. Mater..

[2] Green S., Ferfecki F.J., Marburger U. (2018). Overmoulding of PEEK Compounds for Composites Aerospace Brackets. SAMPE J..

[3] Denault J., Vu-Khanh T. (1992). Crystallization and Fiber/Matrix Interaction During the Molding of PEEK/Carbon Composites. Polym. Compos..

[4] Tierney J.J., Gillespie J.W. (2004). Crystallization kinetics behavior of PEEK based composites exposed to high heating and cooling rates. Compos. A Appl. Sci. Manuf..

[5] Lee A., Wynn M., Quigley L., Salviato M., Zobeiry N. (2022). Effect of temperature history during additive manufacturing on crystalline morphology of PEEK. Adv. Ind. Manuf. Eng..

[6] Wang Y., Chen B., Evans K., Ghita O. (2018). Enhanced Ductility of PEEK thin film with self-assembled fibre-like crystals. Sci. Rep..

[7] Zhang L., Qin Y., Zheng G., Dai K., Liu C., Yan X., Guo J., Shen C., Guo Z. (2016). Interfacial crystallization and mechanical property of isotactic polypropylene based single-polymer composites. Polymer.

[8] Blundell D.J., Osborn B.N. (1983). The morphology of poly(aryl-ether-ether-ketone). Polymer.

[9] Patki R., Mezghani K., Phillips P.J. (2007). Crystallization Kinetics of Polymers. Physical Properties of Polymers Handbook.

[10] Hoffman J.D., Lauritzen J.I. (1961). Crystallization of bulk polymers with chain folding: Theory of growth of lamellar spherulites. J. Res. Natl. Bur. Stand. A Phys. Chem..

[11] Vyazovkin S., Stone J., Sbirrazzuoli N. (2005). Hoffman-Lauritzen parameters for non-isothermal crystallization of poly(ethylene terephthalate) and poly(ethylene oxide) melts. J. Therm. Anal. Calorim..

[12] Xu J., Reiter G., Alamo R.G. (2021). Concepts of nucleation in polymer crystallization. Crystals.

[13] Wang W., Qi Z., Jeronimidis G. (1991). Studies on interface structure and crystal texture of poly(ether-ether-ketone)-carbon fibre composite. J. Mater. Sci..

[14] Karsli N.G., Demirkol S., Yilmaz T. (2016). Thermal aging and reinforcement type effects on the tribological, thermal, thermomechanical, physical and morphological properties of poly(ether ether ketone) composites. Compos. B Eng..

[15] Bas C., Battesti P., Albérola N.D. (1994). Crystallization and melting behaviors of poly(aryletheretherketone) (PEEK) on origin of double melting peaks. J. Appl. Polym. Sci..

[16] Seo J., Zhang X., Schaake R.P., Rhoades A.M., Colby R.H. (2021). Dual Nakamura model for primary and secondary crystallization applied to nonisothermal crystallization of poly(ether ether ketone). Polym. Eng. Sci..

[17] Gordnian K. (2017). Crystallization and Thermo-Viscoelastic Modelling of Polymer Composites. Ph.D. Thesis.

[18] Kong Y., Hay J.N. (2002). Multiple melting behaviour of poly(ethylene terephthalate). Polymer.

[19] Ismail Y.S., Richardson M.O.W., Olley R.H. (2001). Optimizing impact properties of PP composites by control of spherulitic morphology. J. Appl. Polym. Sci..

[20] Motz H. (1987). Characterization of PEEK and Short-Fiber Peek Thermoplastic Composites. Ph.D. Thesis.

[21] Regis M., Bellare A., Pascolini T., Bracco P. (2017). Characterization of thermally annealed PEEK and CFR-PEEK composites: Structure-properties relationships. Polym. Degrad. Stab..

[22] Wynn M., Zobeiry N. (2021). A Fast Method for Evaluating Effects of Process Parameters on Morphology of Semi-Crystalline Thermoplastic Composites. The American Society for Composites—Thirty-Sixth Technical Conference on Composite Materials.

[23] Hayes B.S., Gammon L.M. (2010). Optical Microscopy of Fiber-Reinforced Composites.

[24] Olley R.H., Bassett D.C., Blundell D.J. (1986). Permanganic etching of PEEK. Polymer.

[25] Wang Y., Beard J.D., Evans K.E., Ghita O. (2016). Unusual crystalline morphology of Poly Aryl Ether Ketones (PAEKs). RSC Adv..

[26] Manohar K., Hogan T., Buttrick J., Banerjee A.G., Kutz J.N., Brunton S.L. (2018). Predicting shim gaps in aircraft assembly with machine learning and sparse sensing. J. Manuf. Syst..

[27] Sacco C., Radwan A.B., Anderson A., Harik R., Gregory E. (2020). Machine learning in composites manufacturing: A case study of Automated Fiber Placement inspection. Compos. Struct..

[28] Zobeiry N., Reiner J., Vaziri R. (2020). Theory-guided machine learning for damage characterization of composites. Compos. Struct..

[29] Reiner J., Vaziri R., Zobeiry N. (2021). Machine learning assisted characterisation and simulation of compressive damage in composite laminates. Compos. Struct..

[30] Freed Y., Salviato M., Zobeiry N. (2022). Implementation of a probabilistic machine learning strategy for failure predictions of adhesively bonded joints using cohesive zone modeling. Int. J. Adhes. Adhes..

[31] Freed Y., Zobeiry N., Salviato M. (2022). Development of aviation industry-oriented methodology for failure predictions of brittle bonded joints using probabilistic machine learning. Compos. Struct..

[32] Zobeiry N., Humfeld K.D. (2021). A physics-informed machine learning approach for solving heat transfer equation in advanced manufacturing and engineering applications. Eng. Appl. Artif. Intell..

[33] Kim M., Zobeiry N. Machine Learning for Reduced-order Modeling of Composites Processing. Proceedings of the SAMPE Virtual Conference.

[34] Rasmussen C.E., Williams C.K.I. (2005). Gaussian Processes for Machine Learning.

[35] Liu H., Zhao Y., Li N., Li S., Li X., Liu Z., Cheng S., Wang K., Du S. (2021). Effect of polyetherimide sizing on surface properties of carbon fiber and interfacial strength of carbon fiber/polyetheretherketone composites. Polym. Compos..

[36] Seo J., Gohn A.M., Dubin O., Takahashi H., Hasegawa H., Sato R., Rhoades A.M., Schaake R.P., Colby R.H. (2019). Isothermal crystallization of poly(ether ether ketone) with different molecular weights over a wide temperature range. Polym. Cryst..

[37] Gramacy R.B. (2020). Surrogates: Gaussian Process Modeling, Design, and Optimization.

[38] Himawan C., Starov V.M., Stapley A.G.F. (2006). Thermodynamic and kinetic aspects of fat crystallization. Adv. Colloid Interface Sci..

[39] Varga J., Karger-Kocsis J. (1993). The occurence of transcrystallization or row-nucleated cylindritic crystallization as a result of shearing in a glass-fiber-reinforced polypropylene. Compos. Sci. Technol..

